# Theoretical hydrogen bonding calculations and proton conduction for Eu(iii)-based metal–organic framework†

**DOI:** 10.1039/d1ra01528a

**Published:** 2021-03-22

**Authors:** Lu Feng, Tian-Yu Zeng, Hao-Bo Hou, Hong Zhou, Jian Tian

**Affiliations:** School of Resource and Environmental Science, Wuhan University Wuhan 430072 Hubei China hhb-bhh@163.com; College of Chemistry and Environmental Technology, Wuhan Institute of Technology Wuhan 430073 Hubei China hzhouh@126.com; Hangzhou Yanqu Information Technology Co., Ltd Y2, 2nd Floor, Building 2, Xixi Legu Creative Pioneering Park, No. 712 Wen er West Road, Xihu District Hangzhou City Zhejiang Province 310003 P. R. China

## Abstract

A water-mediated proton-conducting Eu(iii)-MOF has been synthesized, which provides a stable proton transport channel that was confirmed by theoretical calculation. The investigation of proton conduction shows that the conductivity of Eu(iii)-MOF obtained at 353 K and 98% RH is 3.5 × 10^−3^ S cm^−1^, comparable to most of the Ln(iii)-MOF based proton conductors.

## Abstract 2

A water-mediated proton-conducting Eu(iii)-MOF has been synthesized, which provides a stable proton transport channel that was confirmed by theoretical calculation.

## Graphical abstract

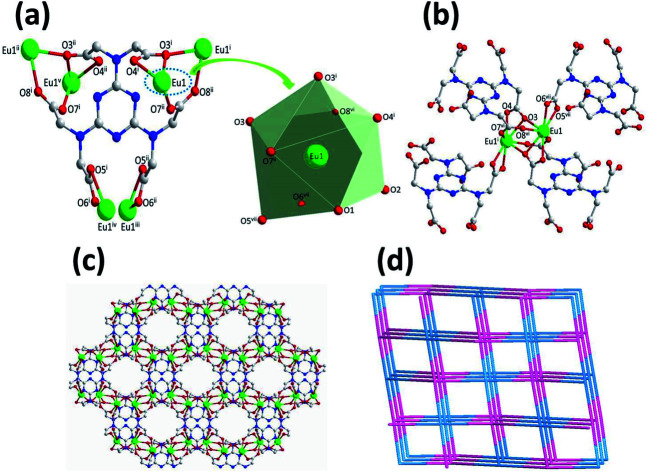

In recent years, with the aggravation of environmental pollution and growing depletion of petroleum, coal and other traditional fossil energy, the demand to exploit alternative cleaner energy is increasingly urgent. Compared to the dispersion of several developed new energy sources, such as solar energy, wind, geothermal heat, and so on,^1^ the proton exchange membrane fuel cell (PEMFC) is recognized as a promising energy conversion system.^2^ As an important component in PEMFC, the proton exchange membrane (PEM) directly affects the transmission efficiency of protons between electrodes.^3^ Currently, Nafion has been widely used as a PEM in commerce, and shows a conductivity higher than 10^−1^ S cm^−1^.^4^ However, the large-scale applications of Nafion are limited due to their high costs, narrow working conditions (low temperature and high relative humidity), amorphous nature, *etc.*^5^ To overcome these limitations, several types of proton-conducting materials have been explored over the past decade.^6^ Among them, MOF materials were employed as ideal platforms to regulate proton conductivity owing to their high crystallinity, tunable structure and tailorable functionality. The crystallographically defined structure is also conductive to the deeply analysis of proton transport path and mechanism,^7^ furthermore, due to the visual structure of MOF, Density Functional Theory (DFT) is recently used to analyse the factors that affect proton conduction from a theoretical perspective, thus providing strong support for the experimental results.^8^ Multi-carboxylate ligands usually exhibit versatile coordination modes and strong complexing ability to metal ions. Moreover, the hydrophilic –COOH groups not only donate protons but also facilitate the formation of continuous hydrogen bond channel with water molecules. Simultaneously, selecting lanthanide metal ions as nodes in the construction of MOF, more water molecules tend to be bound by Ln(iii) ions, leading to an increase in the concentration of proton carrier, which would be beneficial for the effective proton transport. Therefore, the carboxylate-bridged Ln(iii)-MOF are good candidates for proton conduction.^9^ Currently, there are several proton conductive MOF materials, such as {H[(N(Me)_4_)_2_][Gd_3_(NIPA)_6_]}·3H_2_O (*σ* = 7.17 × 10^−2^ S cm^−1^, 75 °C, 98% RH),^10^ Na_2_[Eu(SBBA)_2_(FA)]·0.375DMF·0.4H_2_O (*σ* = 2.91 × 10^−2^ S cm^−1^, 90 °C, 90% RH)^11^ and {[Tb_4_(TTHA)_2_(H_2_O)_4_]·7H_2_O}_*n*_ (*σ* = 2.57 × 10^−2^ S cm^−1^, 60 °C, 98% RH)^12^ that showing ultra-high conductivities (>10^−2^ S cm^−1^). These superprotonic conductors provided advantageous supports for the assembly strategies involved Ln(iii) ion and carboxylate ligand. In this work, 1,3,5-triazine-2,4,6-triamine hexaacetic acid (H_6_TTHA) and Eu(NO_3_)_3_·6H_2_O were assembled at 140 °C for 72 h through solvothermal reaction to afforded colourless crystals, namely {[Eu_2_(TTHA)(H_2_O)_4_]·9H_2_O}_*n*_ (1). This complex has been previously reported by Wu and co-workers.^13^ In their work, the thermal stability and fluorescence properties of 1 were mainly focused. Research suggested that the complex 1 maintained structural stability until 400 °C and demonstrated strong fluorescent emission with high quantum yields (*Φ* > 70%), treating as a good candidate for light applications. To the best of our knowledge, MOFs usually exhibit a variety of potential applications for their structural diversity.^14^ For different researchers, their concerns about the applications of MOF may vary, but it is the continuously exploration and excavation of different performance that will enrich their potentials and meet them in different fields of the applications. Through careful structural analysis, we found that there is a rare infinite water cluster ((H_2_O)_*n*_) existing in the crystal structure of 1 (Fig. S1†), (H_2_O)_*n*_ further interacts with –COO^−^ groups to form an abundant hydrogen bond network (Fig. S2 and Table S1†). The stability of (H_2_O)_*n*_ as well as more complex hydrogen bond formed between (H_2_O)_*n*_ and –COO^−^ groups has been confirmed by the density functional theory (DFT) calculations. The advantageous structural features including high concentration of water molecules and stable hydration channel provide the possibility to realize high proton conductivity of 1. Therefore, the proton conductivities of 1 under varying conditions were investigated in detail.

Complex 1 crystallizes in the monoclinic space group *C*2/*c*, with the asymmetric building unit composed of two Eu(iii) ions, one [TTHA]^6−^ anion, four coordination water molecules and nine lattice water molecules. The Eu(iii) atom is distorted enneahedron coordinated by seven carboxylate oxygen atoms and two water molecules (Fig. 1a and Table S2†). The bond length of Eu–O is in the range of 2.374(5)–2.606(5) Å (Table S3†), comparable to that of the Eu(iii) complex reported in the literature.^15^ The coordination mode of [TTHA]^6−^ can be described as μ_6_-η^2^η^1^η^1^η^1^η^1^η^1^η^1^η^1^η^1^η^1^η^1^η^2^. In the complex 1, the adjacent metal ions were connected through O–C–O and μ–O bridging, forming a dimer, [Eu1]_2_. The dimer acts as a linker and connects with four [TTHA]^6−^ (Fig. 1b). Furthermore, the [TTHA]^6−^ anions coordinate with [Eu1]_2_ through six flexible arms in different directions, leading to the formation of a three-dimensional network structure, where the cavities with regular size of 8.356 × 10.678 Å^2^ are left (Fig. 1c and Fig. S3†). The topological representation of the network of 1 was analysed by using TOPOS software.^16^ As shown in Fig. 1d, the Eu(iii) ions are connected to four [TTHA]^6−^, which can be considered as 4-connected nodes. And the [TTHA]^6−^ anions were also viewed as 4-connected nodes for their connections with four Eu(iii) ions. So, the whole 3D structure was described as a 4,4-*c* net with an extended Schläfli symbol of {4^2^,8^4^}.

**Fig. 1:**
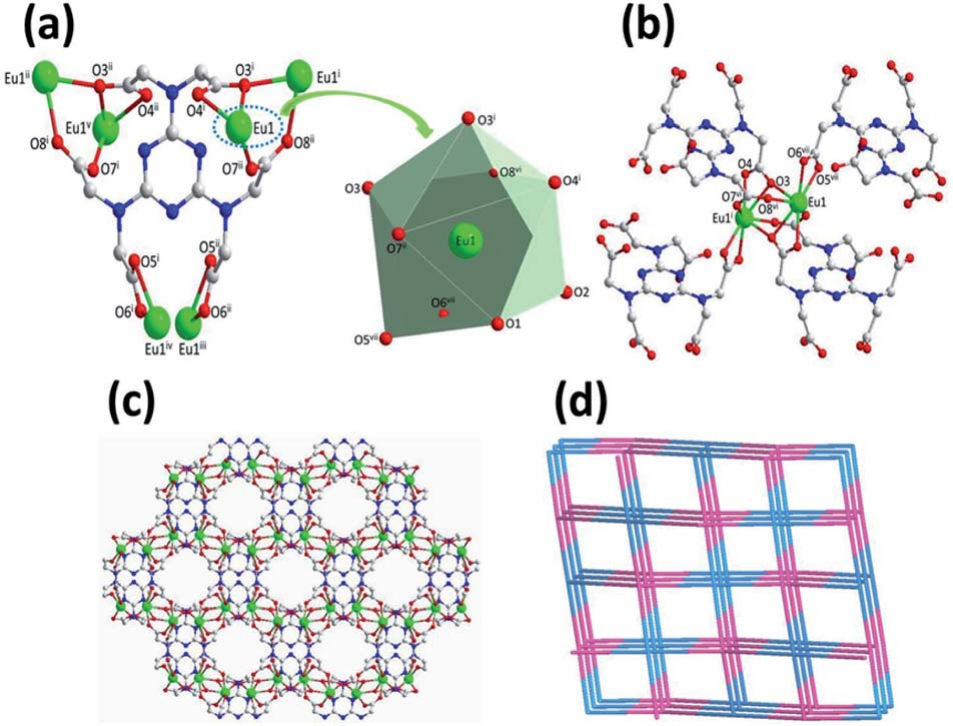
Coordination mode of the [TTHA]^6−^ in 1 showing [EuO_9_] enneahedron (a). The dimer, [Eu1]_2_, formed by O–C–O and μ–O bridging, connects with four [TTHA]^6−^ (b). The 3D structure of 1 formed by the coordination of Eu(iii) and [TTHA]^6−^ as well as water molecules (c). Topological representation of the network of 1 (d). Symmetry codes (i: 1.5 − *x*, 1.5 − *y*, 1 − *z*; ii: 0.5 + *x*, 1.5 − *y*, −0.5 + *z*; iii: *x*, 2 − *y*, −0.5 + *z*; iv: 2 − *x*, 2 − *y*, 1 − *z*; *v*: 2 − *x*, *y*, 0.5 − *z*; vi: 1 − *x*, *y*, 1.5 − *z*; vii: 0.5 + *x*, 0.5 + *y*, *z*).

In 1, the theoretical hydrogen bonding calculations of (H_2_O)_*n*_ and complex cluster were performed using the Gaussian 09 program. All the structures were obtained from the analysis of XRD results and the hydrogen atoms are optimized. We calculated the energy at DFT level by means of B3LYP-D3.^17*a*^ As polarity of molecule has great influence on intermolecular hydrogen bonding,^17*b*^ hydrogen bond-forming orbitals require larger space occupation.^17*c*^ Thus, diffuse and polarization functions augmented split valence 6-311+G(d,p) basis set is used. The binding energy (*E*_binding_) is calculated as the difference between the energy of hydrogen-bonded cluster and the summation of the energies of each component monomer: *E*_binding_ = *E*_tol_ − ∑*N*_i_*E*_i_*E*_tol_ and *E*_i_ are energy of hydrogen-bonded cluster and each individual component monomer, respectively. A hydrogen-bonded cluster is more stable if interaction energy is more negative compared to other hydrogen-bonded configurations. With the help of density functional theory (DFT), we calculate the binding energies (*E*_binding_) to compare the stability of systems. The binding energy of water cluster and complex cluster is −619.65 and −710.34 kcal mol^−1^ (Fig. 2), respectively, indicating the complex cluster system is more stable.

**Fig. 2:**
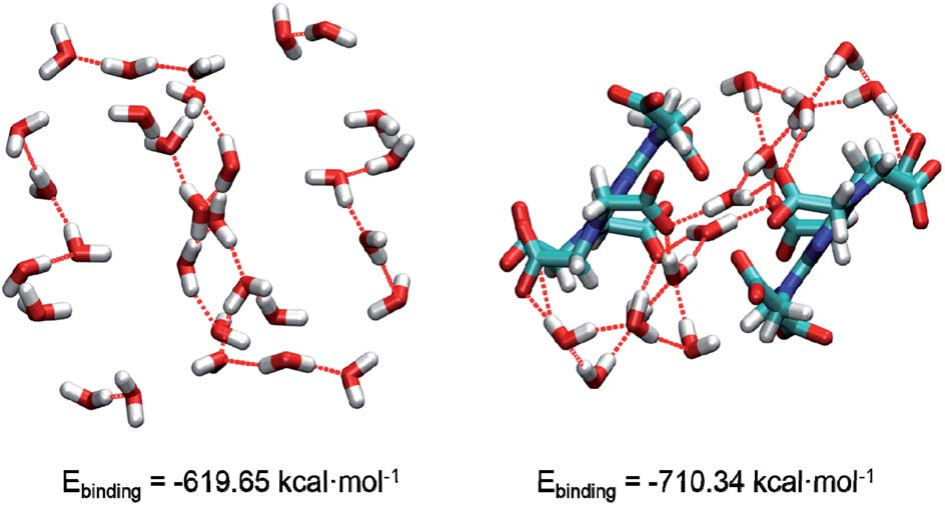
The structures of water cluster and complex cluster. Oxygen, hydrogen, carbon, nitrogen atoms are marked by red, white, cyan, blue, respectively.

The PXRD patterns of 1 were shown in Fig. S4.† It was found that the diffraction peaks of powder sample are in good agreement with the simulated data from single-crystal diffraction, showing the high purity of the synthesized sample. The IR spectrum of 1 exhibits a strong peak at 3422 cm^−1^, which corresponds to the stretching vibration of water molecules.^18*a*^ The absorption peaks appeared at 1551 cm^−1^ and 1400 cm^−1^ are attributed to the antisymmetric stretching of –COO^−^ groups^18*b*^ (Fig. S5†). The water adsorption property of 1 was investigated at 25 °C by DVS Intrinsic Plus. Before the measurement, the sample was treated under 0% RH for 6 h (Fig. S6†). Water adsorption and desorption isotherms of the fully dehydrated sample were shown in Fig. S7.† The adsorption process in the RH range of 0–95% can be divided into three stages. In the initial stage (0–10%), the adsorption of water molecules increased rapidly, which can be attributed to the hydrogen bond interaction between carboxylic acid oxygen atoms and water molecules. Then the water adsorption increased slowly at 10–70% RH, corresponding to the formation of water clusters. Another abrupt increase of water adsorption was found when the RH is above 70%, illustrating that enough energy is needed for the water clusters to exist in the cavity of the crystal.^19^ Clearly, large hysteresis was observed in the adsorption–desorption isotherms, this phenomenon was caused by the strong hydrophilic of –COO^−^ groups in 1.^20^ Furthermore, the structural integrity of the sample after adsorption/desorption cycle was confirmed by PXRD (Fig. S4†).

Based on the previous structural analysis, the proton conduction of 1 was evaluated by the alternating-current (AC) impedance analyses. The Nyquist plots of 1 obtained at different temperature and relative humidity are shown in Fig. 3a and b and Fig. 3d. The resistance is estimated from the intercept of spikes or arcs on the *Z*′ axis, and the conductivity (*σ*) is calculated by the equation of *σ* = *l*/(*A*·*R*), where *l*, *A* and *R* represent the sample thickness, surface area and resistance, respectively. It was found that there are two different modes observed from the impedance spectroscopies under lower relative humidity (60–90% RH), a partial arc at high frequency component can be attributed to the grain interior contribution, while a characteristic spur at low frequency component illustrates that partial-blocking electrode response allows limited diffusion.^21^ So, the only spikes displayed in the Nyquist spectra at 98% RH and 293–353 K suggest that high temperature and high relative humidity are more favourable for the proton conduction. From the temperature-dependent measurements under 98% RH, significantly, the conductivity of 1 increases gradually from 1.34 × 10^−4^ S cm^−1^ at 293 K to 3.5 × 10^−3^ S cm^−1^ at 353 K (Fig. 3c and Table S4†). The increasing conductivity can be attributed to the important role of water molecule. The high concentration of water molecules act as carriers and transmit in the form of H^+^(H_2_O)_*n*_, and the mobility of H^+^(H_2_O)_*n*_ accelerates with the rising temperature. Moreover, the higher acidity of water molecules at higher temperature is more conducive to the improvement of proton conductivity. The relative humidity dependence measured at 298 K indicated that the conductivity of 1 presented significant positive correlations with the humidity changes. The conductivity is 1.42 × 10^−5^ S cm^−1^ at 60% RH and increases to be 1.63 × 10^−4^ S cm^−1^ at 98% RH (Fig. 3e and Table S5†). This can be explained by the ability of (H_2_O)_*n*_ to bind water molecules and strong hydrophilic of –COO^−^ group that has been confirmed by the water adsorption process, especially when the RH is above 60%. For water-mediated proton conductors, the lower RH usually results in the insufficient of transport media and further affects the diffusion of protons. At present, the theoretical simulations (*e.g.* aMS-EVB3)^22^ and activation energy (*E*_a_)^23–27^ are the main methods to analysis the proton conduction mechanism. Compared with the theoretical calculations, the judgment rule with *E*_a_ is more straightforward. Here, the *E*_a_ of 1 determined from the linear fit of ln(*σT*) *vs.* 1000/*T* is 0.44 eV (Fig. 3f), which reveals that the proton transfer in 1 follows a typical vehicle mechanism.^12^ Further evaluate the long-term stability of 1, the time-dependent proton conductivity has been conducted, indicating negligible decline of proton conductivities even lasted 12 h (Fig. 4, S8 and Table S6†). The sample of 1 after property measurements was collected and characterized by PXRD to examine any structural change, and the PXRD spectrum shows structural integrity even at high temperature and high relative humidity environment (Fig. S4†). The long-term stable proton conductivities of 1 can be attributed to the robust hydrogen bonding channel that has been confirmed by the DFT calculations. In recent years, the proton conductive carboxylate-based MOF have been systematic reviewed by G. Li’ group,^9^ it was found that the complex 1 shows higher conductivity of 3.5 × 10^−3^ S cm^−1^ under 353 K and 98% RH when compared to the Ln(iii)-MOF materials, such as [Me_2_NH_2_][Eu(ox)_2_(H_2_O)]·3H_2_O (*σ* = 2.73 × 10^−3^ S cm^−1^, 95% RH, 55 °C),^23^ {[Gd(ma)(ox)(H_2_O)]_*n*_·3H_2_O} (*σ* = 4.7 × 10^−4^ S cm^−1^, 95% RH, 80 °C),^24^ (N_2_H_5_)[Nd_2_(ox)_4_(N_2_H_5_)]·4H_2_O (*σ* = 2.7 × 10^−3^ S cm^−1^, 100% RH, 25 °C),^25^ {[SmK(BPDSDC)(DMF)(H_2_O)]·*x*(solvent)}_*n*_ (*σ* = 1.11 × 10^−3^ S cm^−1^, 98% RH, 80 °C),^26^ [Nd(mpca)_2_Nd(H_2_O)_6_Mo(CN)_8_]·*n*H_2_O (*σ* = 2.8 × 10^−3^ S cm^−1^, 98% RH, 80 °C),^27^ MFM-550(M) and MFM-555(M) (M = La, Ce, Nd, Sm, Gd, Ho) (*σ* = 1.46 × 10^−6^ to 2.97 × 10^−4^ S cm^−1^, 99% RH, 20 °C)^28^ as well as other conductive materials showing lower conductivities in the range of 10^−9^ to 10^−5^ S cm^−1^.^9^ However, the conductivity of 1 is inferior to those Ln(iii)-MOFs with conductivities higher than 10^−2^ S cm^−1^ × ^10–12^ In recent years, another two H_6_TTHA-derived MOF and CP, {[Tb_4_(TTHA)_2_(H_2_O)_4_]·7H_2_O}_*n*_^12^ and {[Co_3_(H_3_TTHA)_2_(4,4′-bipy)_5_(H_2_O)_8_]·12H_2_O}_*n*_^19*b*^ have been previously reported by our group, which show highest proton conductivities of 2.57 × 10^−2^ S cm^−1^ at 60 °C and 8.79 × 10^−4^ S cm^−1^ at 80 °C under 98% RH, respectively. The noticeable performance difference between these two complexes and 1 was analysed based on the visual structures. The higher conductivity of 1 when compared to the Co(ii) complex can be attributed to the concentration of water molecules, 23.25% for 1 and 15.92% for the Co(ii) complex. The high concentration of proton carrier in 1 promotes the transfer of protons. Although the water molecular concentration of 1 is higher than that of the Tb(iii) complex, however, the coordination numbers of Ln(iii) ions in the two compounds are different, eight for the Tb(iii) ion and nine for the Eu(iii) ion, respectively. The coordination sites are obviously not satiated, especially for the Tb(iii) complex, the lower coordination number may prone to chelate more water molecules under high relative humidity, leading to the formation of more consecutive hydration channel with TTHA^6−^ anions and water molecules, thus accelerating the proton transport. In contrast, the molecular structure of the Eu(iii) compound contains nearly a quarter of water molecules, these water molecules have almost filled the pores, so the smaller pore structure is difficult to accommodate more adsorbed water molecules.

**Fig. 3:**
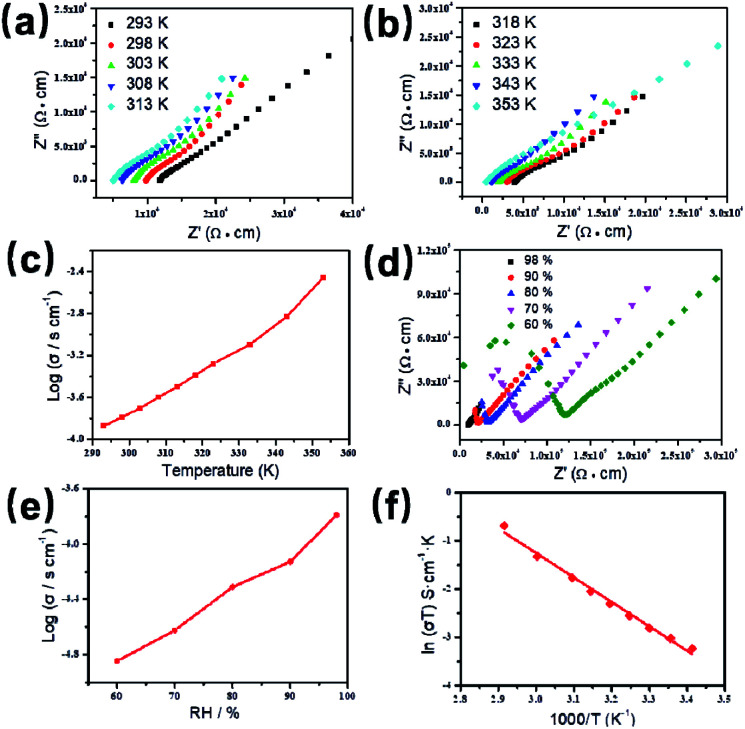
Nyquist plots for proton conductivity of 1 (98% RH) at 293–313 K (a) and 318–353 K (b). Plot of log(*σ*) *vs. T* for 1 in the temperature range of 293–353 K (c). Plots of the impedance plane for 1 at different relative humidities and 298 K (d). Humidity dependence of the proton conductivity at 298 K (e). Arrhenius plot of 1 at 98% RH (f).

**Fig. 4:**
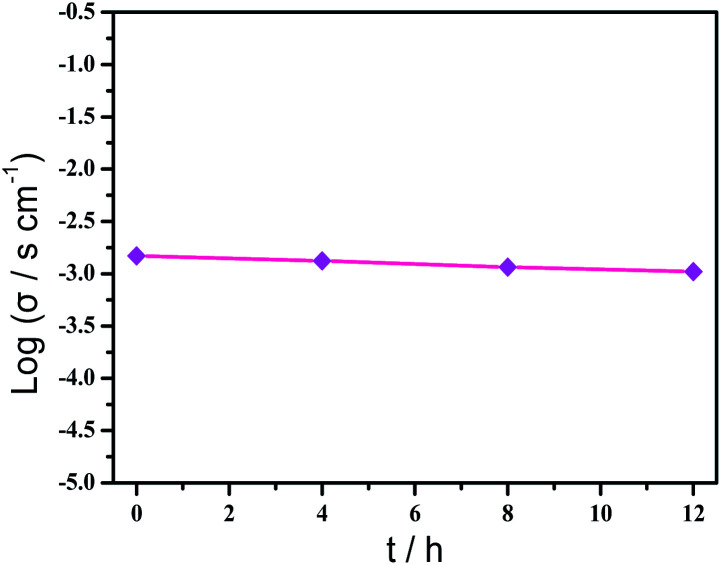
Time-dependent proton conductivity of 1 at 343 K and 98% RH.

In conclusions, a water-mediated proton-conducting Eu(iii)-MOF has been synthesized, displaying a 3D network structure with high concentration water molecules and –COO^−^ groups as well as abundant H-bond networks. Interesting, there is an infinite water cluster of (H_2_O)_*n*_ existing in the crystal structure of Eu(iii)-MOF, which is rare in the H_6_TTHA-derived complexes and even other reported MOF/CPs. Based on this, the density functional theory was conducted to evaluate the stability of water cluster and complex cluster. As expected, the calculated binding energies indicate that the more stable system was formed by (H_2_O)_*n*_ and –COO^−^ groups, which provides a favourable guarantee for proton conduction. The advantageous structural features of Eu(iii)-MOF result in the realization of comparable proton conductivity of 3.5 × 10^−3^ S cm^−1^ at 353 K and 98% RH and long-term stability at least 12 h. Additionally, the factors affecting the electrical conductivity of several H_6_TTHA-derived MOF/CPs have been compared and analysed from the visual structures, and the structure-activity relationship of such compounds was also summarized, which will provide guidance to design novel crystalline superprotonic conductors assembled from multi-carboxylate.

## Conflicts of interest

There are no conflicts to declare.

## Supplementary Material

RA-011-D1RA01528A-s001

RA-011-D1RA01528A-s002
