# Supplementary material for: Theoretical hydrogen bonding calculations and proton conduction for Eu(iii)-based metal–organic framework
Source: RSC Adv. 2021 Mar 22;11(19):11495–9. doi: 10.1039/d1ra01528a (PMC8698213; doi:10.1039/d1ra01528a)
Supplement: RA-011-D1RA01528A-s001 [file RA-011-D1RA01528A-s001.pdf]

Electronic Supplementary Material (ESI) for RSC Advances.
This journal is © The Royal Society of Chemistry 2021

# Theoretical hydrogen bonding calculations and proton conduction for Eu(III)-based metal-organic framework

**Lu Feng,[a] Tian-Yu Zeng,[a] Hao-Bo Hou,*[a] Hong Zhou*[b] and Jian Tian[c]**

[a.] *School of Resource and Environmental Science, Wuhan University, Wuhan 430072, Hubei, China. Corresponding author: hhb-bhh@163.com*

[b.] *College of Chemistry and Environmental Technology, Wuhan Institute of Technology, Wuhan 430073, Hubei, China. Corresponding author: hzhouh@126.com*

[c.] *Hangzhou Yanqu Information Technology Co., Ltd., Y2, 2nd Floor, Building 2, Xixi Legu Creative Pioneering Park, No. 712 Wen' er West Road, Xihu District, Hangzhou City, Zhejiang Province, 310003, P.R.O.C.*

# Supporting Information

# Experimental

## 1. Materials

Chemical reagents were purchased commercially and used as received without further purification. The ligand of 1,3,5-triazine-2,4,6-triamine hexaacetic acid ($H_6$TTHA) was prepared according to the method reported in literature. [1]

## 2. Physical measurements

Elemental analysis (C, H and N) was performed by using a Perkin-Elmer 2400 series II CHN analyzer. The powder X-ray diffraction measurement was conducted on a Bruker D8 ADVANCE X-ray diffractometer. Infrared (IR) spectrum was recorded in the range 4000 - 400 $cm^{-1}$ on a FT-IR analyzer (1601, shimadzu Co., Japan) by using KBr-pellet method. The water adsorption property of **1** was investigated at 25 $^0$C and in the RH range of 0%-95% by DVS Intrinsic Plus (Surface Measurement Systems, English). Before the measurement, the sample was treated under 0% RH for 6 h until the water molecules in the samples were completely removed.

## 3. Synthesis of the complex $\{[Eu_2(TTHA)(H_2O)_4]\cdot 9H_2O\}_n$ (1)

$H_6$TTHA (0.04 mmol, 0.0191 g), 4, 4'-bipy (0.12 mmol, 0.019 g), $Eu(NO_3)_3\cdot 6H_2O$ (0.04 mmol, 0.0181 g), 2 mL water and 1 mL acetonitrile were added in 10 mL vial. Then, 90 µL 6 mol/L HCl was added. The vial was kept in an autoclave at 140 $^0$C for three days. Colorless crystals were obtained and washed with deionized water. Yield: 63 % (35.41 mg, based on Eu). Anal. Calcd. For $C_{15}H_{38}Eu_2N_6O_{25}$: C, 17.9; H, 3.81; N, 8.35 %. Found: C, 17.54; H, 3.49; N, 8.76 %. Main IR data (KBr, $cm^{-1}$): 3422(m), 3267(m), 2941(w), 1551(s), 1492(m), 1432(w), 1400(m), 1306(s), 1192(m).

## 4. X-ray crystallography

Single-crystal X-ray diffraction data for **1** was collected at 173 K on a Bruker Smart CCD area-detector diffractometer with graphite-monochromatic Mo/Kα radiation (λ = 0.71073 Å) in ω-scan mode. The collected data were reduced using the software package SAINT [2] and semi-empirical absorption correction was applied to the intensity data using SADABS program. [3] The structure of **1** was solved using direct methods, and all nonhydrogen atoms were refined anisotropically by least squares on F2 using the SHELXTL-2014 program.4 Hydrogen atoms were placed in calculated positions and refined isotropically using the riding model. Details of the hydrogen-bond geometry, crystallographic data and selected bond lengths (Å) for **1** are summarized in Table S1, Table S2 and Table S3, respectively.

## 5. Proton conductivity studies

Electrical characterization was carried out on a cylindrical pellet (10 mm of diameter and 0.5 mm of thickness) obtained by pressing 50 mg of sample at 500 MPa for 5 min. The pellet was pressed between porous C electrodes (Sigracet, GDL 10 BB, no Pt). Impedance spectroscopy data were collected using a HP4284A impedance analyzer over the frequency range from 20 Hz to 1 MHz with an applied voltage of 0.2 V. Electrical measurements were taken at different temperature (293 - 353 K) and relative humidity (60%, 70%, 80%, 90% and 98%) as well as different times (0h, 4h, 8h and 12h). All measurements were electronically controlled by the winDETA package of programs. [5]

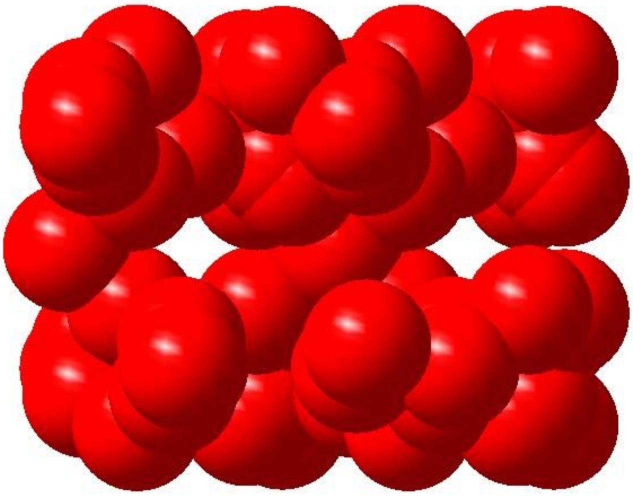

**Fig. S1**The space-filling model of water cluster (($(H_2O)_n$).

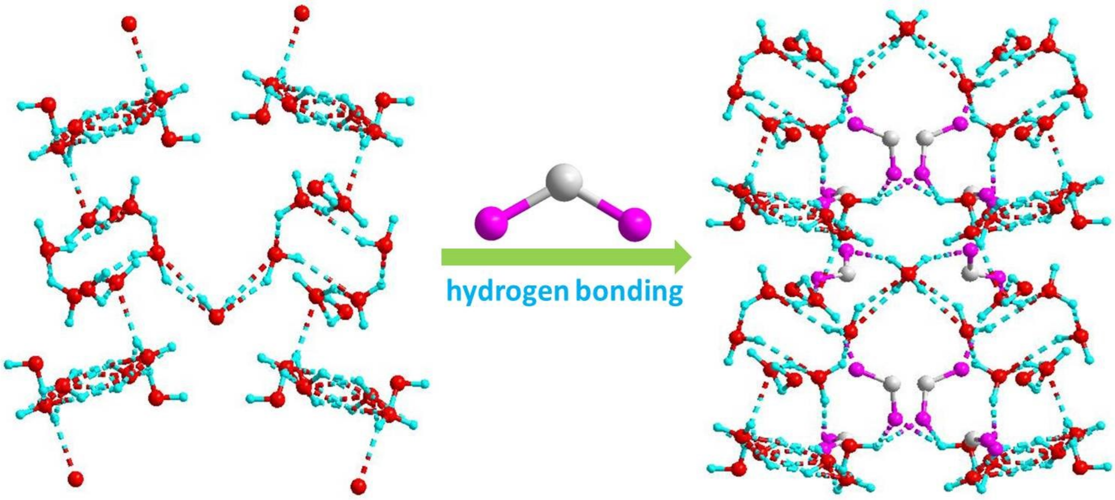


**Fig. S2** The infinite water cluster of $(H_2O)_n$ (left) and abundant hydrogen bond network formed by $(H_2O)_n$ and –$COO^-$ groups (right), where the carboxylic acid oxygen atoms are labeled as pink for clearly.

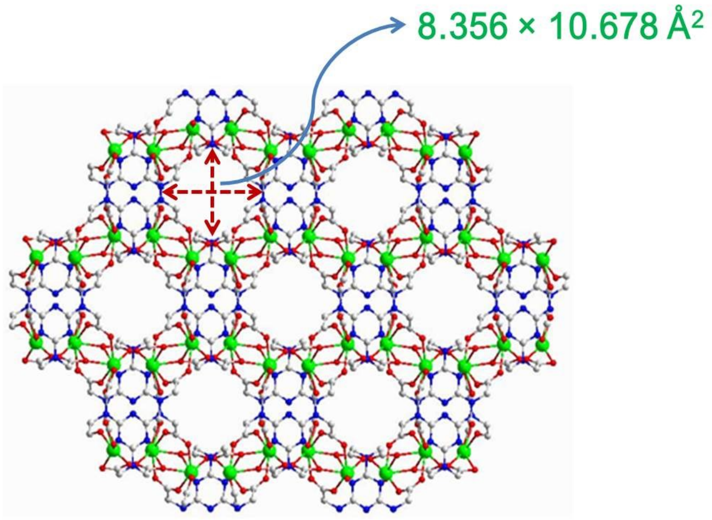


**Fig. S3** The cavities with regular size of 8.356 × 10.678 Å$^2$ are left in the three-dimensional network structure of **1**.

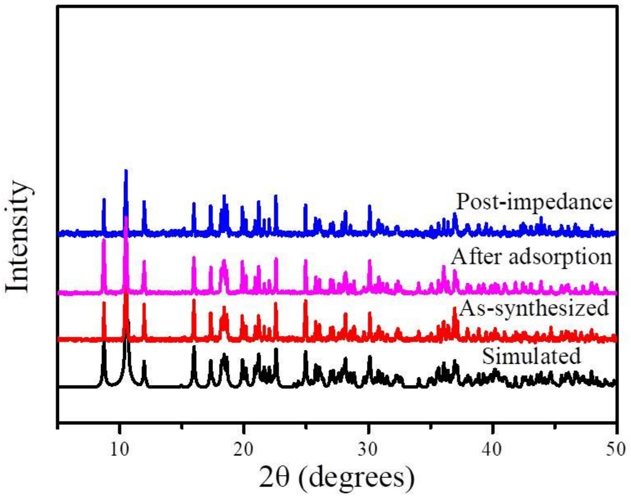


**Fig. S4** The XRD patterns of **1** under different experimental conditions.

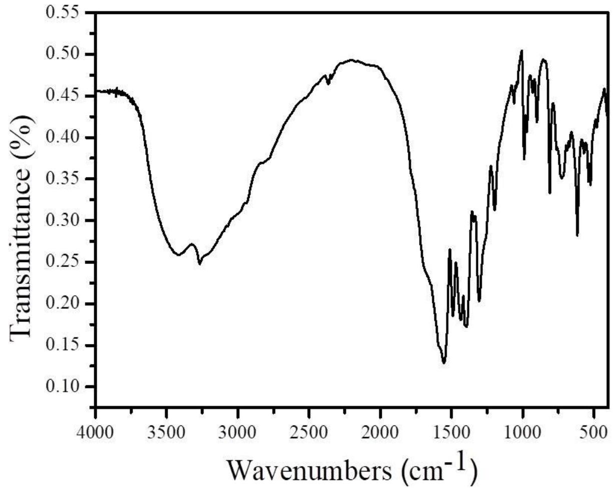


**Fig. S5** The IR spectrum of **1**.

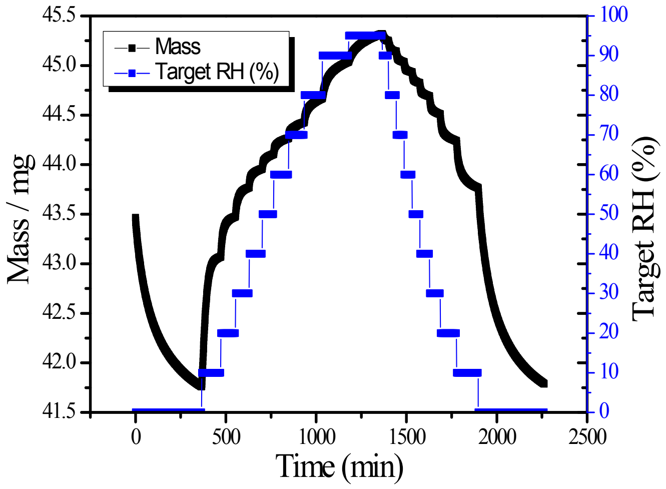


**Fig. S6** The change curves of mass and RH with time for the complex **1**.

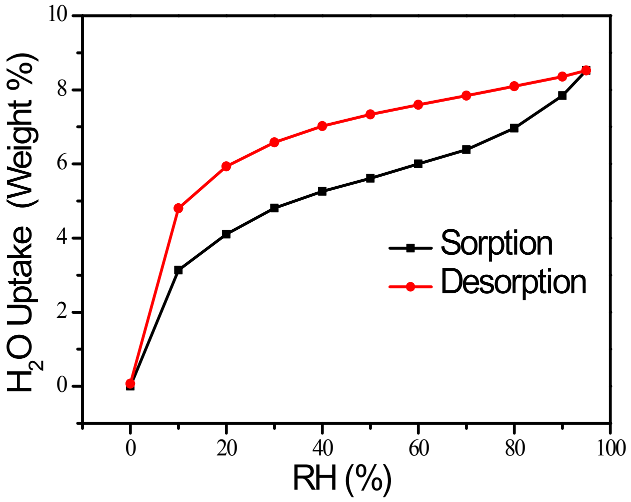


**Fig. S7** Water adsorption–desorption isotherms (25 $^0$C) of **1** measured by DVS Intrinsic Plus.

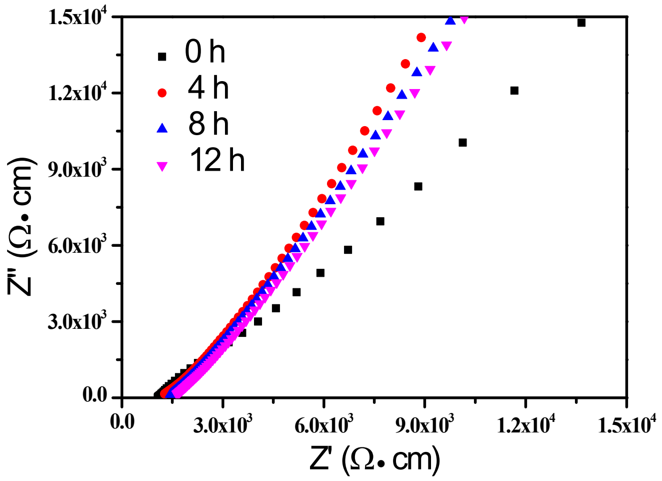


**Fig. S8** The Nyquist plots for proton conductivity of **1** (343 K and 98% RH) at 0h, 4h, 8h and 12h.

**Table S1** Hydrogen-bond geometry (Å, °) for **1**.

| D-H···A | D-H | H···A | D···A | D-H···A |
|---|---|---|---|---|

| | | | | |
|---|---|---|---|---|
| O1-H1A···O7$^{i}$ | 0.876(6) | 2.320(5) | 2.713(7) | 107.4(4) |
| O1-H1B···O9$^{ii}$ | 0.876(5) | 2.044(6) | 2.701(8) | 131.1(4) |
| O9$^{ii}$-H9A$^{ii}$···O5$^{ii}$ | 0.871(6) | 1.941(5) | 2.737(8) | 151.3(4) |
| O9$^{ii}$-H9B$^{ii}$···O10$^{iii}$ | 0.871(7) | 2.084(7) | 2.863(9) | 148.5(5) |
| O13$^{i}$-H13A$^{i}$···O13A$^{i}$ | 0.870(2) | 1.642(2) | 0.818(3) | 13.0(2) |
| O13$^{i}$-H13B$^{i}$···O9$^{ii}$ | 0.871(2) | 1.990(5) | 2.554(2) | 121.4(1) |
| O13A$^{i}$-H13C$^{i}$···O13$^{i}$ | 0.870(2) | 1.522(2) | 0.818(3) | 24.8(1) |
| O13A$^{i}$-H13D$^{i}$···O13$^{i}$ | 0.869(2) | 1.306(2) | 0.818(3) | 37.9(2) |
| O13A$^{i}$-H13D$^{i}$···O13$^{iV}$ | 0.869(2) | 2.507(2) | 2.888(3) | 107.3(1) |
| O13A$^{i}$-H13D$^{i}$···O13A$^{iV}$ | 0.869(2) | 1.742(2) | 2.193(3) | 109.6(1) |
| O10$^{iii}$-H10A$^{iii}$···O4$^{iii}$ | 0.870(7) | 2.219(5) | 3.031(8) | 155.4(5) |
| O10$^{iii}$-H10B$^{iii}$···O12$^{v}$ | 0.870(7) | 2.244(7) | 2.726(9) | 114.9(5) |
| O12$^{v}$-H12A$^{v}$···O11$^{vi}$ | 0.868(7) | 2.033(6) | 2.786(1) | 144.4(5) |
| O12$^{v}$-H12B$^{v}$···O10$^{vii}$ | 0.869(8) | 2.363(7) | 3.106(1) | 143.7(5) |
| O11$^{vi}$-H11A$^{vi}$···O12$^{vi}$ | 0.870(6) | 2.075(7) | 2.786(1) | 138.3(2) |
| O11$^{vi}$-H11B$^{v}$···O8$^{viii}$ | 0.869(6) | 1.999(5) | 2.804(6) | 153.7(1) |
| O2$^{ix}$-H2A$^{ix}$···O13$^{vii}$ | 0.877(5) | 2.353(2) | 2.813(2) | 112.9(6) |
| O2$^{ix}$-H2A$^{ix}$···O13A$^{vii}$ | 0.877(5) | 2.399(2) | 2.730(2) | 102.7(6) |
| O2$^{ix}$-H2B$^{ix}$···O6$^{ii}$ | 0.876(6) | 2.000(5) | 2.684(7) | 134.1(4) |
| O2$^{ix}$-H2B$^{ix}$···O6$^{i}$ | 0.876(6) | 2.418(5) | 2.810(7) | 107.6(4) |

**Symmetry codes (i: 0.5+x, 1.5-y, -0.5+z; ii: 1.5-x, 1.5-y, 1-z; iii: 1+x, y, z; iv: 2-x, y, 1.5-z; v: 2.5-x, 0.5+y, 1.5-z; vi: 0.5+x, 0.5+y, z; vii: 2-x, 2-y, 1-z; viii: 1.5+x, 0.5+y, z; ix: x, 2-y, -0.5+z).**

**Table S2** Crystallographic data and refinement parameters of **1**

| | **1** |
|---|---|
| CCDC | 2036641 |
| Empirical formula | $C_{15}H_{38}Eu_2N_6O_{25}$ |
| Formula weigh | 1006.43 |
| Temperature/K | 173 |
| Crystal system | monoclinic |
| Space group | *C* 2/*c* |
| a/Å | 12.7130(11) |
| b/Å | 16.7745(15) |
| c/Å | 14.7977(13) |
| α/° | 90 |
| β/° | 91.265(4) |
| γ/° | 90 |
| V/Å³ | 3154.9(5) |
| Z | 4 |

| | |
|---|---|
| $D_{calc}$/g cm$^{-3}$ | 2.119 |
| μ/mm$^{-1}$ | 4.048 |
| F(000) | 1984.0 |
| h, k, l max | 15, 20, 17 |
| No. of parameters | 243 |
| S | 0.998 |
| $R_1$, $wR_2$ [I > 2σ(I)] | 0.0392, 0.07 |
| $\Delta\rho_{max}$ and $\Delta\rho_{min}$, e Å | 0.745, 0.566 |

**Table S3** Selected bond lengths (Å) of **1**.

| **1** | | | |
|---|---|---|---|
| Eu1-O1 | 2.472(5) | Eu1-O2 | 2.424(5) |
| Eu1-O3$^{i}$ | 2.606(5) | Eu1-O4$^{i}$ | 2.478(6) |
| Eu1-O3 | 2.374(5) | Eu1-O5$^{ii}$ | 2.524(5) |
| Eu1-O6$^{ii}$ | 2.461(5) | Eu1-O7$^{iii}$ | 2.401(5) |
| Eu1-O8$^{iv}$ | 2.379(5) | | |

**Symmetry codes (i: 1.5-x, 1.5-y, 1-z; ii: 0.5+x, 0.5+y, z; iii: 0.5+x, 1.5-y, -0.5+z; iv: 1-x, y, 1.5-z)**

**Table S4** The resistance (R) and conductivity (σ) of **1** under different temperature and 98% RH. The values of pellet dimensions including sample thickness (*l*) and diameter are 500 um and 2 mm, respectively.

| Temperature (K) | R (Ω) | σ (S/cm) |
|---|---|---|
| 293 | 11892.8 | $1.34 \times 10^{-4}$ |
| 298 | 9785.52 | $1.63 \times 10^{-4}$ |
| 303 | 8025.75 | $1.98 \times 10^{-4}$ |
| 308 | 6321.61 | $2.52 \times 10^{-4}$ |
| 313 | 5016.63 | $3.17 \times 10^{-4}$ |
| 318 | 3914.39 | $4.07 \times 10^{-4}$ |
| 323 | 3013.24 | $5.28 \times 10^{-4}$ |
| 333 | 1980.25 | $8.04 \times 10^{-4}$ |
| 343 | 1074.69 | $1.48 \times 10^{-3}$ |
| 353 | 455.31 | $3.50 \times 10^{-3}$ |

**Table S5** The resistance (R) and conductivity (σ) of **1** under different relative humidity and 298 K.

| RH (%) | R (Ω) | σ (S/cm) |
|---|---|---|
| 60 | 112098.428 | $1.42 \times 10^{-5}$ |
| 70 | 66985.6459 | $2.38 \times 10^{-5}$ |
| 80 | 32516.53 | $4.9 \times 10^{-5}$ |
| 90 | 21360.46 | $7.45 \times 10^{-5}$ |
| 98 | 9785.52 | $1.63 \times 10^{-4}$ |

**Table S6** The resistance (R) and conductivity (σ) of **1** (343 K and 98% RH) under different time.

| Time (h) | R (Ω) | σ (S/cm) |
|---|---|---|
| 0 | $1.07 \times 10^3$ | $1.48 \times 10^{-3}$ |
| 4 | $1.12 \times 10^3$ | $1.43 \times 10^{-3}$ |
| 8 | $1.38 \times 10^3$ | $1.16 \times 10^{-3}$ |
| 12 | $1.52 \times 10^3$ | $1.05 \times 10^{-3}$ |

## References


1 P. de Hoog, P. Gamez, W. L. Driessen and J. Reedijk, *Tetrahedron Lett.,* 2002, **43**, 6783.

2 M. Er, R. Ustabaş, U. Coruh, K. Sancak, E. Vázquez-López, *Int. J. Mol. Sci.,* 2008, **9**, 1000.

3 G. M. Sheldrick, SADABS: Program for Empirical Absorption Correction of Area Detector Data, University of Göttingen, Germany, 1996.

4 G. M. Sheldrick, SHELX2013, Programs for Crystal Structure Analysis; Institüt für Anorganische Chemie der Universität: Göttingen, Germany, 1998.

5 *winDETA*, Novocontrol GmbH: Hundsangen, Germany, 1995.
